# Revascularization of Coronary Artery Chronic Total Occlusion by Active Antegrade Reverse Wire Technique

**DOI:** 10.1155/2021/8893946

**Published:** 2021-02-10

**Authors:** Xiao-jiao Zhang, Zhan-xiu Zhang, Yong Wang, Pei-pei Hou, Da-ming Mu, Cheng-fu Wang, De-feng Luo, Bao-jun Chen, Ai-jie Hou, Bo Luan

**Affiliations:** ^1^Department of Cardiology, The People's Hospital of China Medical University, The People's Hospital of Liaoning Province, Shenyang, China; ^2^Department of Cardiology, The Second People's Hospital of Huludao, Huludao, China

## Abstract

**Objectives:**

To assess the effectiveness and safety of ARW for vascular recanalization in CTO patients.

**Background:**

Chronic total occlusion (CTO) of coronary artery accompanied with large branch distal to the occluded segment (<2 mm) is one of the challenges physicians are facing during the coronary intervention. In cases where the antegrade wire passed the occluded segment reaching the branch vessel, but could not access the main vessel through various adjustments, application of active antegrade reverse wire technique (ARW) could be considered. *Patients and Methods*. A total of 301 consecutive CTO patients who received the antegrade percutaneous coronary intervention (PCI) between December 2015 and December 2019 at our institution were included, of whom 11 were treated with ARW (10 successfully) for vascular recanalization. The applicability and safety of ARW were assessed.

**Results:**

Among the 301 CTO patients who received antegrade vascular recanalization, 11 were treated with ARW. ARW was successful in 10 patients as follows: from the diagonal branch (D) to anterior descending branch (LAD) in 4 patients; from the septal branch (S) to LAD in 1 patient; from D to S and LAD in 1 patient; from the circumflex branch (LCX) to obtuse marginal branch (OM) in 1 patient; from OM to LCX in 1 patient; from a posterior descending artery (PDA) to the posterior lateral vein (PLV) in 2 patients. Yet, ARW in patient with RCAm CTO failed, while the consequent retrograde PCI succeeded. The mean J-CTO score of the 11 patients was 2.7 ± 0.65, among whom eight were accompanied with calcifications. Sion Black and Fielder XTR reverse wires were used in 9 and 2 patients, respectively. No loss of side branches or severe procedure-related complications occurred in 11 patients.

**Conclusion:**

Therefore, ARW can improve procedural efficiency and should be popularized for further application.

## 1. Introduction

**Figure 1:**
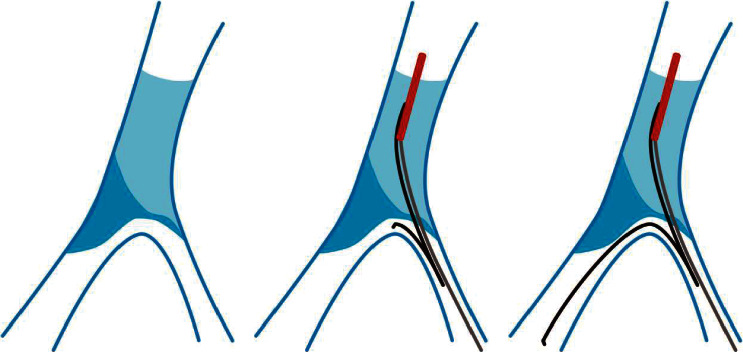
Antegrade recanalization of CTO by ARW included three steps as follows: (1) after the wire accessed the branch, angiography through microcatheter was conducted to distinguish true lumen from false lumen; (2) a double cavity microcatheter and a hydrophilic-coated wire ((a) 90° shaping at 1 mm to the headend; (b) reflexed shaping at 2-3 cm to the headend) were concurrently delivered, and a small balloon could also be used to dilate the access with low pressure (avoiding changing the structures of the bifurcation) (b); and (3) withdrawal of the double cavity microcatheter, while the wire was adjusted to access the true lumen of the segment distal to the occlusion (c).

Currently, chronic total occlusion (CTO) is considered as the most challenging condition in coronary percutaneous coronary intervention. Despite the rapid advancement of the techniques and equipment for CTO-PCI that has occurred over the past decade, the success rate of antegrade recanalization of CTO still reaches only about 60%–80% [1]. When accompanied with relatively large branch vessels distal to the occluded segment, especially when the branches are close to the occluded segment (<2 mm), the antegrade wiring could be difficult to perform due to the influences from the structures of tissues, which in some cases could even cause the damage to the crest of branches or loss of side branches. Such cases are very difficult to perform. If the occluded segment is accompanied by calcification or fibrous plaques, the proximal and distal segments of the occlusion showed an h- or reversed h-shaped morphology (Figure 1(a)). Consequently, it is difficult to deliver the wire to the landing zone, which could increase the procedural time and X-ray exposure and even induce coronary dissection, dilation of false lumen, loss of side branches, or pericardial tamponade. In some patients, the wire could also be used to access the side branches through the “relative weak points.” Therefore, the conventional reverse wire technique (RW) [2] that is used in patients with coronary bifurcation lesions should be changed to active antegrade reverse wire technique (ARW), which could increase the success rate of the procedure and reduce the X-ray exposure in antegrade recanalization of CTO.

## 2. Materials and Methods

### 2.1. Technical Description

ARW was applied for the CTO patients accompanied with distal bifurcation, defined as relatively large branch (diameters ≥1.5 mm) at the distal ostial of the occluded segment (≤2 mm), while there maybe a microaccess between the branch and occluded segment. In such patients, the formation and texture of the plaques in the occluded segment (calcification plaques or heavier fibrous plaques) could influence the structure of the intersection of bifurcation shown as h- or reversed h-shape, which facilitate the access of the wire to the branch not to the distal segment of the occlusion (Figures 1(a)–1(c)).

### 2.2. Study Population

ARW was applied in the procedural of antegrade recanalization in CTO patients in People's Hospital of Liaoning Province between December 2015 and December 2019. All the procedures were conducted by Dr. Bo Lan (the corresponding author of this article). For all the patients, loading doses of aspirin (300 mg) and clopidogrel (300 mg) (or 180 mg ticagrelor) were administered before the procedure. Afterward, dual antiplatelet therapy, including 100 mg aspirin (oral, once per day) and 75 mg clopidogrel (oral, once per day) (or 90 mg ticagrelor, oral, twice per day), was applied. The access route was mainly right radial artery or right femoral artery, while other access could also be selected if necessary, according to the operators' decisions. During the procedure, standard dose of heparin (100 IU/kg) was used for anticoagulation, with 2000 IU regular heparin being added every hour to maintain ACT at 300–350 s. This study was approved by the Institutional Review Board on Ethics of People's Hospital of Liaoning Province. The study abided by the Declaration of Helsinki and all the patients signed informed consent before participating in the study.

## 3. Results

A total of 301 CTO patients receiving antegrade PCI were included in the study, of whom 11 received ARW for the recanalization of CTO. The mean J-CTO score of the patients was 2.7 ± 0.65, and 8 of them were accompanied by calcification. In detail, ARW was conducted from diagonal branch (D) to anterior descending branch (LAD) in 4 patients; from the septal branch (S) to LAD in 1 patient; from D to S and LAD in 1 patient; from the circumflex branch (LCX) to obtuse marginal branch (OM) in 1 patient; from OM to LCX in 1 patient; and from a posterior descending artery (PDA) to the posterior lateral vein (PLV) in 2 patients; the treatment was successful in all these 10 patients (residual stenosis of 0% and blood flow of TIMI grade 3). However, the treatment in patient with RCAm CTO failed. In this case, however, after the Fielder XTR wire was delivered to PDA, the adjustment of Sion Black wire to PLV failed and retrograde PCI was successfully performed. Polymer-jacket hydrophilic-coated wire was used as the reverse wire in the ARW for all the 11 patients, among whom Sion Black wire and Fielder XTR wire were used for 9 and 2 patients, respectively. No side branch loss or procedural-related complications occurred. The procedural data are shown in Table 1.

## 4. Two Cases Received ARW

**Figure 2:**
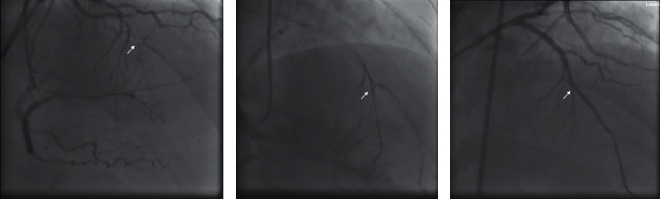
(a) A patient with CTO at the proximal anterior descending branch (LAD), where the distal end of the occluded segment was blunt, and with a relatively large septal branch. The true opening of the main vessel was h-shaped due to the restriction of the plaques. (b) ARW was used to reversely deliver the wire from the septal branch to LAD. (c) The occluded segment was successfully recanalized.

The first case was a proximal LAD-CTO lesion with J-CTO score 3 points (the previous recanalization failed; with blunt proximal cap and mild calcification), and the length of the occluded segment was <20 cm. Bilateral angiography showed a blunt bifurcation coronary lesion at the distal ostial of the occluded segment. At the same time, there was a relatively large septal branch distal to the occluded segment. The ostial of the main vessel was h-shaped due to the restriction of the plaques, which was also the cause of recanalization failure in the other hospital (Figure 2(a)).

In this study, antegrade 7F EBU 3.75 Guiding was used in the procedure, and Fielder XTR wire was used to access the middle segment of the occlusion after supported by using the OTW balloon, which was then changed to Miracle 3 wire. However Miracle 3 wire was difficult to cross the occlusion of the main coronary artery, the wire was adjusted to the septal branch, and then we changed Miracle to Sion Blue wire in septal branch. The Sion Black wire was 90° bend shaped at 1 mm of the tip outside the body and reflex-shaped at 3 cm, which was then entering into septal branch through the KDL microcatheter. The Sion Black wire was advanced successfully into the true lumen of distal LAD (Figure 2(b)), and then PCI was successfully conducted in the LAD-CTO (Figure 2(c)).

## 5. Discussion

In the present study, we described ARW, a new technique for antegrade recanalization of CTO, and further demonstrated the safety of this technique. About 26.4% to 30% of CTO patients tend to present with coronary bifurcation lesions, among which 52% are CTO with distal bifurcation. These, in turn, may increase the procedural difficulties and complications, thus reducing the success rate [3, 4]. If the occluded segment accompanied by calcification or fibrous plaques, the proximal and distal segments of the occlusion showed an h- or reversed h-shaped morphology. The angle between the proximal end of occlusion and branch was close to 180°, which could easily lead the wire to the branch rather than the main coronary artery, especially when there were calcifications or relatively severe fibrous plaques in proximal main coronary artery [5, 6]. Yet, it is challenging to advance the wire to the distal landing zone. We speculated that the reverse wire technique could apply in markedly angulated bifurcated lesions in CTO lesions [7], which had high effectiveness in reversely advancing the wire from the main vessel to branches. Therefore, we applied ARW for the recanalization of CTO, utilizing the special structures to recanalize the main vessel from branches. This technique not only increased the success rate of the procedure without increasing the procedure-related complications but also maximally preserved the branches.

**Figure 3:**
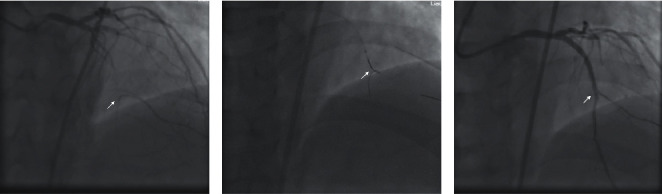
The second case was a patient with CTO at proximal LAD, in whom the J-CTO score was 3 points (the previous recanalization failed; with mild calcification and length >20 mm). There were a lot of branches at the opening, and the length of the occluded segment was relatively long. Retrograde angiography showed blunt bifurcation at the opening of the occluded segment, while the diagonal branch (D) and LAD showed an h-shape (a). In the present study, antegrade 7F EBU 3.75 Guiding was used for the surgery, and Fielder XTR wire was used to deliver the 135 cm Corsair (Asahi Intecc ) wire to the diagonal branch and then was replaced with Sion Blue wire. The headend of the Sion Black wire was 90° shaped at 1 mm to the headend outside the body and reflex shaped at 4 cm, which was then delivered to the diagonal branch through the KDL double cavity microcatheter. The Sion Black wire was adjusted and successfully delivered to the true lumen at distal LAD (b), after which the CTO in LAD was successfully recanalized, and a stent was implanted (c).

Ten of eleven patients received successful recanalization of CTO lesions using ARW technique from large branch (including D, S, OM, PLV, and PDA) to main coronary artery. There were no branch losses or procedure-related complications in the present study. The patient in whom ARW failed was a mid-right coronary artery CTO. The patient had one PLVost-p stent implanted before. In this study, however, several attempts of reverse delivery of Sion Black wire from PDA to PLV failed. We speculated that the ridge of PLVost stent might prevent the wire from passing through, thus leading to the failure of ARW. The consequent retrograde PCI from septal to PLV was successfully carried out (Figure 3).

We summarized the clinical situation for ARW technique: (1) the antegrade wire was in the true lumen all the time and was directly advanced into the distal branch, which was the safest situation. (2) The antegrade wire was advanced to the subintimal track before the bifurcation and then passed through the proximal ridge of the branch into the true lumen, which was a relatively safe situation. (3) The antegrade wire was delivered to the false lumen before the bifurcation, which passed through the distal ridge of the branch and traveled for certain distances and then into the true distal lumen. In this situation, the wire could be reversely delivered to the distal end of the occlusion. However, due to the artificial false lumen, the risk of branch loss was very high after stent implantation, and the incidence of perioperative acute myocardial infarction could be increased; in addition, this could influence the long-term survival [8]. In such cases, ARW should not be applied, and revascularization of branches should be sufficiently prepared in special cases. An intravascular ultrasound examination could also be conducted to clarify the route of the wire into side branches, which could facilitate the application of ARW.

Following issues should be considered during the ARW: (1) during the advancing of microcatheter or low-pressure dilation of small balloon, the microchannel between the main vessel and branch should not be destroyed if possible. (2) According to our experience, the polymer-jacket hydrophilic-coated wire should be chosen for ARW if possible, as the ultralubricated coat could favor the wire to assess the main vessel using ARW, which is in line with the previous study by Nomura et al. [9]. The shaping of the tip of the reverse wire should be conducted according to the lesions. In the present study, we mainly used 1 mm 90° bend in the tip of the wire, as our experience from various attempts showed that such bend could best favor the advancing of the guidewire. (3) During the procedure, the cooperativity and necessary concordance should be maintained among guiding, branch wire, dual lumen microcatheter, and reverse wire, while the deep insertion of guiding should be avoided.

In summary, ARW is an effective technique, which is not only safe but can also increase the efficiency of antegrade interventional therapy. These findings need to be further validated in clinical practice.

## 6. Limitations

This study was a single-center study by the same operators. The sample size was relatively small. The operators had learning curves, and thus the success rate of the procedures between December 2015 and December 2019 increased yearly with an increasing number of cases and the equipment advances, which could also influence the findings. More multicenter studies with a larger sample size conducted by more experienced operators are needed to validate our findings.

## 7. Conclusion

ARW is an effective technique, which is not only safe but can also increase the efficiency of antegrade interventional therapy. These findings need to be further validated in clinical practice.

## Figures and Tables

**Table 1:** Cases using ARW technique for CTO-PCI.

	Case1	Case2	Case3	Case4	Case5	Case6	Case7	Case8	Case9	Case10	Case11
Sex	M	M	M	F	M	M	F	M	M	F	M
Age	57	60	40	59	61	68	64	73	78	60	54
CTO Vessel	LADp	LADp	LADp	LADp	LADp	LADp	LCXm	LCXm	RCAm	RCAp	RCAm
J-CTO	3	3	3	2	2	4	2	2	3	3	3
MC/OTW	Corsair-KDL	OTW-KDL	Corsair-KDL	Corsair-KDL	Corsair-KDL	Corsair-KDL	Corsair-KDL	Corsair-KDL	Corsair-KDL	Corsair-KDL	Corsair-KDL
SB-MB	D-LAD	S-LAD	D-LAD	D-LAD	D-LAD	D-S-LAD	LCXm-OM	OM-LCXm	PDA-PLV	PDA-PLV	PDA-PLV
The wire to SB	Conquest Pro	Miracle 3	Fielder-XTR	Gaia 2	Conquest Pro	Conquest Pro	Conquest Pro	Gaia 2	Fielder-XTR	Gaia 3	Conquest Pro
Reverse wiring	Fielder-XTR	Sion black	Sion black	Fielder-XTR	Sion black	Sion black	Sion black	Sion black	Sion black	Sion black	Sion black
Lesion calcification	Yes	Yes	No	Yes	Yes	Yes	Yes	Yes	No	No	Yes
Reverse Wiring Success	Yes	Yes	Yes	Yes	Yes	Yes	Yes	Yes	No	Yes	Yes
Lost SB	No	No	No	No	No	No	No	No	No	No	No
Procedural Success	Yes	Yes	Yes	Yes	Yes	Yes	Yes	Yes	Yes (Retrograde)	Yes	Yes
Contrast volume(ml)	300	280	320	210	220	500	260	240	280	260	200
Fluoroscopy time(min)	85	70	106	60	65	161	75	65	110	80	70
Antegrade Guiding	7F EBU3.5	7F EBU3.75	7F EBU3.75	7F EBU3.5	7F EBU3.75	7F EBU3.5	7F EBU3.5	7F EBU3.75	7F JR3.5	7F AL1	7F JR4.0

## Data Availability

The data supporting the results in the current study are available from the corresponding author upon reasonable request.
